# Thromboembolic adverse events associated with TPO-RA in ITP treatment: a pharmacovigilance analysis of the FDA Adverse Event Reporting System

**DOI:** 10.3389/fimmu.2026.1669486

**Published:** 2026-02-13

**Authors:** Zhen Lu, Yingjian Zeng, Guangbin Shang, Xiaonan Lu

**Affiliations:** 1 Jiangxi University of Chinese Medicine, Nanchang, China; 2 The Affiliated Hospital of Jiangxi University of Chinese Medicine, Nanchang, China; 3 Research Center for Differentiation and Development of Basic Theory of Traditional Chinese Medicine, Nanchang, China; 4 School of Chinese Medicine, Jiangxi University of Chinese Medicine, Nanchang, China

**Keywords:** adverse events, FAERS, immune thrombocytopenia, pharmacovigilance, TPO-RA

## Abstract

**Background:**

Thrombopoietin receptor agonists (TPO-RA) are widely used for immune thrombocytopenia (ITP), but their post-marketing thromboembolic safety profiles and onset patterns remain incompletely characterized.

**Methods:**

FAERS reports from January 2009 to December 2024 were cleaned to remove duplicates and non-suspected roles, yielding 2,092 unique thromboembolic AE cases. TPO-RA exposure was identified by generic and brand names. Disproportionality analyses employed reporting odds ratio (ROR), proportional reporting ratio (PRR), and Bayesian confidence propagation neural network (BCPNN). Time-to-onset analysis and logistic regression (univariate and multivariate) examined demographic and treatment factors.

**Results:**

Avatrombopag, Eltrombopag, and Romiplostim yielded 105, 1,044, and 943 thromboembolic AE reports, respectively. Pulmonary embolism, deep vein thrombosis, and portal vein thrombosis predominated with Avatrombopag; Eltrombopag and Romiplostim were mainly associated with pulmonary embolism, deep vein thrombosis, and acute myocardial infarction. Avatrombopag showed the strongest ROR signal for renal vein thrombosis (ROR = 136.49; 95% CI: 56.53–329.56), while Eltrombopag and Romiplostim exhibited highest signals for renal embolism (ROR = 23.70; 95% CI: 8.78–64.00) and arterial embolism (ROR = 37.63; 95% CI: 25.68–55.14), respectively. Median time to onset was 81 days (IQR: 25–263), with 25% of events occurring within 25 days. Multivariate analysis identified age > 85 years (OR = 14.94; 95% CI: 12.51–17.97), body weight > 100 kg (OR = 1.43; 95% CI: 1.26–1.63), and treatment duration > 730 days (OR = 1.32; 95% CI: 1.21–1.45) as independent factors associated with increased reporting odds of thromboembolic events. Compared with Avatrombopag, Eltrombopag (OR = 0.32; 95% CI: 0.25–0.40) and Romiplostim (OR = 0.20; 95% CI: 0.16–0.25) were associated with lower reporting odds of thromboembolic AE.

**Conclusions:**

Real-world pharmacovigilance evidence indicates that thromboembolic event reporting for TPO-RAs shows drug-specific patterns, with venous events predominating and a notable fraction occurring early after treatment initiation. Patients of very advanced age, those with higher body weight, and those receiving prolonged therapy appear particularly vulnerable, supporting proactive baseline risk stratification and sustained thrombosis surveillance throughout treatment.

## 1 Background

Immune thrombocytopenia (ITP) is an acquired autoimmune disorder characterized by isolated thrombocytopenia (platelet count <100×10^9/L) caused by immune-mediated platelet destruction and impaired platelet production. It is a diagnosis of exclusion, established after ruling out other causes of thrombocytopenia (e.g., drug-induced thrombocytopenia, infections, bone marrow disorders, thrombotic microangiopathies, and consumptive coagulopathies). The primary clinical signs are petechiae and ecchymoses on the skin and mucous membranes, as well as spontaneous hemorrhaging. The incidence in adults was reported to be 1.6–3.9/10^5^ annually, with a prevalence rate of up to 9.5/10^5^. A female predominance is observed in the younger population, whereas gender distribution appears more balanced in the elderly group (1). Persistent thrombocytopenia not only increases the risk of life-threatening bleeding and intracranial hemorrhage but is also associated with fatigue, decreased quality of life, and overall mortality. The underlying pathophysiology is based on the “dual disorder of destruction and production” by anti-platelet antibodies and cell-mediated immunity, involving several pathways such as FcγR-mediated splenic phagocytosis, complement-dependent lysis, impaired megakaryocyte maturation, and Treg dysfunction (2, 3). The old treatment modalities of the 20th century were mostly glucocorticoids, intravenous immunoglobulin, and splenectomy. However, these methods were limited by suboptimal response rates and elevated relapse rates. Since 2008, the approval of TPO-RAs (Avatrombopag, Eltrombopag, and Romiplostim) has significantly altered the framework of ITP therapy.

Although the benefits of TPO-RA in the treatment of ITP have been fully confirmed, there have been reports suggesting a potential risk of thrombosis associated with its use. A meta-analysis of randomized controlled trials shows that the overall incidence of thromboembolic events associated with TPO-RA is 2–6%, and cases occurring within ≤3 months after exposure indicate the existence of susceptible individuals (4). Long-term follow-up of clinical trials and real-world reports have documented thromboses of unusual venous sites such as portal vein, renal vein, deep vein-pulmonary artery, and mesenteric vein thrombosis, which are accompanied by arterial complications including myocardial infarction or stroke (5, 6). In a large open-label extension study, the exposure-adjusted thrombosis rate is approximately 5.5/100-years, with the main types being Deep Vein Thrombosis, Pulmonary Embolism, and Myocardial Infarction (7). The thromboembolic risk in ITP patients treated with TPO-RA exhibits a significant time dependence: the incidence of thrombosis in the short term (≤12 weeks) is minimal and does not significantly differ from that of the control group. As treatment duration extends, there is an increase in the occurrence of thromboembolic events. The overall incidence of thrombosis is approximately 6% after a median treatment duration of 2.4 years, with an increasing proportion of arterial thrombosis. Several factors significantly increase thromboembolic risk, including elevated platelet peaks, sustained high platelet counts, or splenectomy (8, 9).

The FDA Adverse Event Reporting System (FAERS), as the world’s largest real-world pharmacovigilance database, plays a central role in drug safety surveillance research (10–12). The FAERS database allows researchers to efficiently identify associations between different drugs and adverse events (AEs), serving as a crucial foundation for improving drug safety monitoring and clinical risk assessment (13, 14). Currently, the FAERS database has demonstrated unique advantages in monitoring thromboembolic AEs, particularly in the systematic evaluation of large-scale real-world evidence (15, 16). As the principal data platform endorsed by the FDA for post-marketing drug safety monitoring, FAERS integrates comprehensive case reports from various populations and regions globally, covering multiple reporting sources such as healthcare professionals, patients, and pharmaceutical companies. This database is particularly useful for identifying thromboembolic events with a low incidence. This approach identifies thromboembolic risk signals that may not be evident in clinical trials and facilitates a comprehensive analysis of event timing, risk factors, and vulnerable populations (17–19).

Based on these established methodologies, this study aims to systematically evaluate thromboembolic AEs associated with TPO-RA using the FAERS database, thereby providing real-world evidence to inform clinical decision-making about the safety of TPO-RA.

## 2 Methods

### 2.1 Data sources

The data for this study were obtained from the publicly accessible FAERS database, which aggregates spontaneous AE reports from multiple sources, thereby providing insight into AE reporting patterns and safety signals. We selected the FAERS dataset as the primary data source for this analysis, encompassing reports from January 2009 to December 2024. All AEs were coded using Preferred Terms (PT) according to the Medical Dictionary for Regulatory Activities (MedDRA, version 24.0). All PTs indicative of symptoms, signs, and potentially associated examinations can be classified into narrative categories utilizing Standardized MedDRA Queries (SMQ) to delineate the medical conditions of interest. After removing 3,262,004 duplicate records per FDA guidelines, key fields such as PRIMARYID, CASEID, and FDA_DATE were extracted. For reports sharing the same CASEID, only the entry with the latest FDA_DATE was retained; if both CASEID and FDA_DATE were identical, the record with the highest PRIMARYID was retained. Time-to-onset (TTO) was calculated as the interval between TPO-RA initiation and AE onset (TTO = AE onset date − TPO-RA initiation date) and was assessed only in reports with complete, valid dates for both fields; 912 reports had calculable TTO and were included in the time-to-onset analysis. Reports with negative TTO values, incomplete dates (missing day/month/year), or implausible date sequences (i.e., the event date preceding the start date) were excluded. In this study, thromboembolic AEs were classified into 3 categories of narrow SMQ.

**Figure 1:**
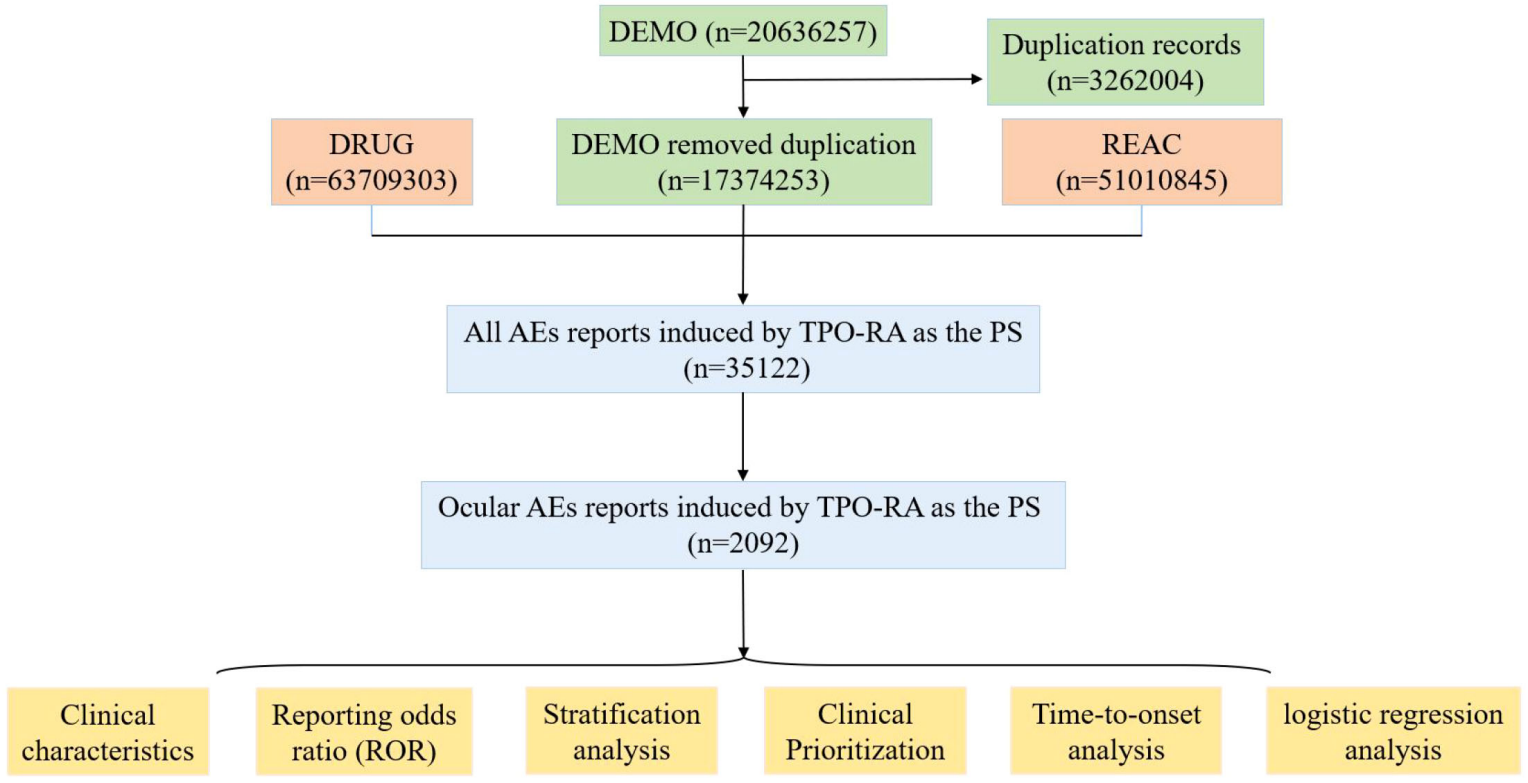
The process of selecting AEs associated with TPO-RA from FAERS and the research workflow.

In the FAERS database, individual case reports (representing unique patients) may contain multiple associated AE reports. Therefore, the following data cleaning procedures were implemented (**Figure 1**): (1) Duplicate reports (the same case/report submitted from different sources) and multiple cases/reports (follow-ups to the same case/report containing additional and updated information) were removed. Only the latest version of each unique case report was retained to eliminate duplicates; (2) Only AE cases/reports where the reported drug role was “suspected” were included, while those with “concomitant” or “interacting” roles were excluded; (3) Our analysis finally concentrated on three FDA-approved TPO-RAs: Avatrombopag, Eltrombopag, and Romiplostim. Both generic names and brand names were used to identify records of thromboembolic events associated with TPO-RAs; (4) The role for TPO-RA was assigned by the reporters using specific role codes, such as preferred suspect (PS), secondary suspect (SS), concomitant (C), and interacting (I). As a spontaneous reporting database, FAERS is subject to confounding and reporting biases, including indication-related confounding, stimulated/notoriety reporting, and residual duplicate reports. We restricted the primary analysis to reports where the TPO-RA was coded as PS to reduce misattribution from concomitant therapies and to improve the specificity of drug–event associations in a spontaneous reporting setting. After implementing the duplicate removal and screening processes, a total of 2,092 thromboembolic AE reports related to TPO-RAs were identified for further analysis (Avatrombopag n=105; Eltrombopag n=1,044; Romiplostim n=943).

FAERS variables such as age, sex, and body weight are incompletely reported. For descriptive summaries, proportions were calculated using the number of reports with non-missing values for the corresponding variable (available-case analysis), and the denominator is reported where relevant. For regression analyses, reports with missing values in model covariates were excluded from that specific analysis (complete-case analysis for the multivariable model).

### 2.2 Statistical analysis

We employed Reporting Odds Ratio (ROR), Proportional Reporting Ratio (PRR), and Bayesian Confidence Propagation Neural Network (BCPNN) methods to identify AE signals associated with the target drugs. The PRR and χ² were calculated based on the 2x2 contingency table for performing disproportionality analysis. If all three methods yielded positive results, it could be inferred that the proposed criteria were satisfied, thus categorizing the problematic signals as suspected AE signals (20, 21) (**Tables 1**, **2**). Variables demonstrating statistical significance in the univariate logistic regression analysis were subsequently incorporated into the multivariate logistic regression study. Univariate logistic regression analyses were performed for age, weight, and drugs. Variables with a p-value < 0.05 were included in the multivariate logistic regression analysis. Fisher’s exact test was used for small sample sizes or when the expected frequency was less than 5, while the χ² test was applied to large samples with an expected frequency of at least 5 in each cell. Data were analyzed and graphed using R software (version 4.3.0). Base R functions were used together with a custom in-house R package (developed by the authors) for data cleaning, deduplication, and dataset management. Data visualization was performed using the ggplot2 package. A p-value < 0.05 was considered statistically significant.

**Table 1:** Three major algorithms are used for signal detection.

Algorithms	Calculation formula	Threshold
ROR	$\begin{array}{c} ROR=\frac{\left(a/c\right)}{\left(b/d\right)}=\frac{ad}{bc}\\ SE(InROR)=\sqrt{\left(\frac{1}{a}+\frac{1}{b}+\frac{1}{c}+\frac{1}{d}\right)}\\ 95\%CI=eIn(ROR)\pm 1.96\sqrt{\left(\frac{1}{a}+\frac{1}{b}+\frac{1}{c}+\frac{1}{d}\right)} \end{array}$	a≥3 with a lower 95% CI > 1
PRR	$\begin{array}{c} PRR=\frac{a/(a+b)}{c/(c+d)}\\ SE(InPRR)=\sqrt{\frac{1}{a}-\frac{1}{a+b}+\frac{1}{c}-\frac{1}{c+d}}\\ 95\%CI=eIn(PRR)\pm 1.96\sqrt{\frac{1}{a}-\frac{1}{a+b}+\frac{1}{c}-\frac{1}{c+d}} \end{array}$	a≥3 with a lower 95% CI > 1
BCPNN	$\begin{array}{c} IC=\log_{2}\frac{p(x,y)}{p(x)p(y)}=\log_{2}\frac{a(a+b+c+d)}{\left(a+b)(a+c\right)}\\ E(IC)=\text{lo}{\text{g}}_{2}\frac{\left(\text{a}+\text{γ}11)(\text{a}+\text{b}+\text{c}+\text{d}+\text{α})(\text{a}+\text{b}+\text{c}+\text{d}+\text{β}\right)}{\left(\text{a}+\text{b}+\text{c}+\text{d}+\text{γ})(\text{a}+\text{b}+\text{α}1)(\text{a}+\text{b}+\text{β}1\right)}\\ V(IC)=\frac{1}{{\left(In2\right)}^{2}}\left\{\left[\frac{(\text{a}+\text{b}+\text{c}+\text{d})-\text{a}+\text{γ}-\text{γ}11}{\left(\text{a}+\text{γ}11)(1+\text{a}+\text{b}+\text{c}+\text{d}+\text{γ}\right)}\right]+\left[\frac{(\text{a}+\text{b}+\text{c}+\text{d})-(\text{a}+\text{b})+\text{α}-\text{α}1}{(\text{a}+\text{b}+\text{c}+\text{d})-(\text{a}+\text{b})+\text{α}-\text{α}1}\right]+\left[\frac{(\text{a}+\text{b}+\text{c}+\text{d})-(\text{a}+\text{c})+\text{β}-\text{β}1}{\left(\text{a}+\text{c}+\text{β}1)(1+\text{a}+\text{b}+\text{c}+\text{d}+\text{β}\right)}\right]\right\}\\ \gamma =\gamma 11\frac{\left(\text{a}+\text{b}+\text{c}+\text{d}+\text{α})(\text{a}+\text{b}+\text{c}+\text{d}+\text{β}\right)}{\left(\text{a}+\text{b}+\text{α}1)(\text{a}+\text{c}+\text{β}1\right)}\\ IC-2SD=E(IC)-2\sqrt{v(IC)} \end{array}$ α1=β1 = 1; α=β=2; γ11 = 1	(-): E(IC)≤0 (+):0< E(IC) ≤1.5: (++):1.5< E(IC) ≤3: (+++):E(IC)>3

a, number of reports containing both the target drug and target adverse drug reaction; b, number of reports containing other adverse drug reaction of the target drug; c, number of reports containing the target adverse drug reaction of other drugs; d, number of reports containing other drugs and other adverse drug reactions. 95%CI, 95% confidence interval; N, the number of reports; χ2, chi-squared; IC, information component; IC025, the lower limit of 95% CI of the IC; E(IC), the IC expectations; V(IC), the variance of IC.

**Table 2:** Two-by-two contingency table for disproportionality analyses.

	Target AEs	Other AEs	Total
Target drugs	a	b	a+b
Other drugs	c	d	c+d
Total	a+c	b+d	a+b+c+d

AEs, adverse events; a, number of reports containing both the target drug and target adverse drug reaction; b, number of reports containing other adverse drug reaction of the target drug; c, number of reports containing the target adverse drug reaction of other drugs; d, number of reports containing other drugs and other adverse drug reactions.

## 3 Results

### 3.1 Demographic information

We extracted and analyzed thrombosis-related case reports associated with Avatrombopag, Eltrombopag, and Romiplostim, yielding totals of 105, 1,044, and 943 cases, respectively. The male-to-female ratios for each drug were 62.9% vs 33.3%, 52.4% vs 39.7%, and 42.4% vs 42.4%, respectively. Among the 1,465 reports with age available, the 18–65 years age group represented the largest proportion, comprising 37.1%, 36.0%, and 33.1%, respectively. A total of 622 patients provided weight data, with the group of body weight 50–100 kg accounting for the highest proportion, at 14.3%, 21.1%, and 25.9% respectively. Case reports originating from the United States represented the largest proportions, at 31.4%, 34.1%, and 49.0%. (**Table 3**).

**Table 3:** Basic characteristics of patients with AEs associated with TPO-RA from the FAERS database.

Characteristics	Avatrombopag	Eltrombopag	Romiplostim
Gender (n, %)			
Female	66 (62.9%)	547 (52.4%)	400 (42.4%)
Male	35 (33.3%)	414 (39.7%)	400 (42.4%)
NA	4 (3.8%)	83 (8.0%)	143 (15.2%)
Age (n, %)			
<18	4 (3.8%)	27 (2.6%)	10 (1.1%)
18-65	2 (1.9%)	40 (3.8%)	52 (5.5%)
65-85	39 (37.1%)	376 (36.0%)	312 (33.1%)
>85	28 (26.7%)	296 (28.4%)	279 (29.6%)
NA	32 (30.5%)	305 (29.2%)	290 (30.8%)
Weight (n, %)			
<50 kg	1 (1.0%)	39 (3.7%)	24 (2.5%)
>100 kg	3 (2.9%)	35 (3.4%)	41 (4.3%)
50–100 kg	15 (14.3%)	220 (21.1%)	244 (25.9%)
NA	86 (81.9%)	750 (71.8%)	634 (67.2%)
Country (n, %) (The top 5 are listed in descending order of the total number of reports)			
United States	33 (31.4%)	356 (34.1%)	462 (49.0%)
Italy	17 (16.2%)	39 (3.7%)	38 (4.1%)
Spain	8 (7.6%)	35 (3.4%)	44 (4.6%)
Germany	6 (5.7%)	45 (4.3%)	43 (4.5%)
United Kingdom	6 (5.7%)	26 (2.5%)	44 (4.6%)
Reporter role			
Consumer	21 (20.0%)	269 (25.8%)	72 (7.6%)
Health Professional	19 (18.1%)	76 (7.3%)	88 (9.3%)
Pharmacist	2 (1.9%)	54 (5.2%)	36 (3.8%)
Physician	61 (58.1%)	466 (44.6%)	567 (60.1%)
NA	2 (1.9%)	179 (17.1%)	180 (19.1%)

### 3.2 Analysis of AE signals at the PT level

**Figure 2:**
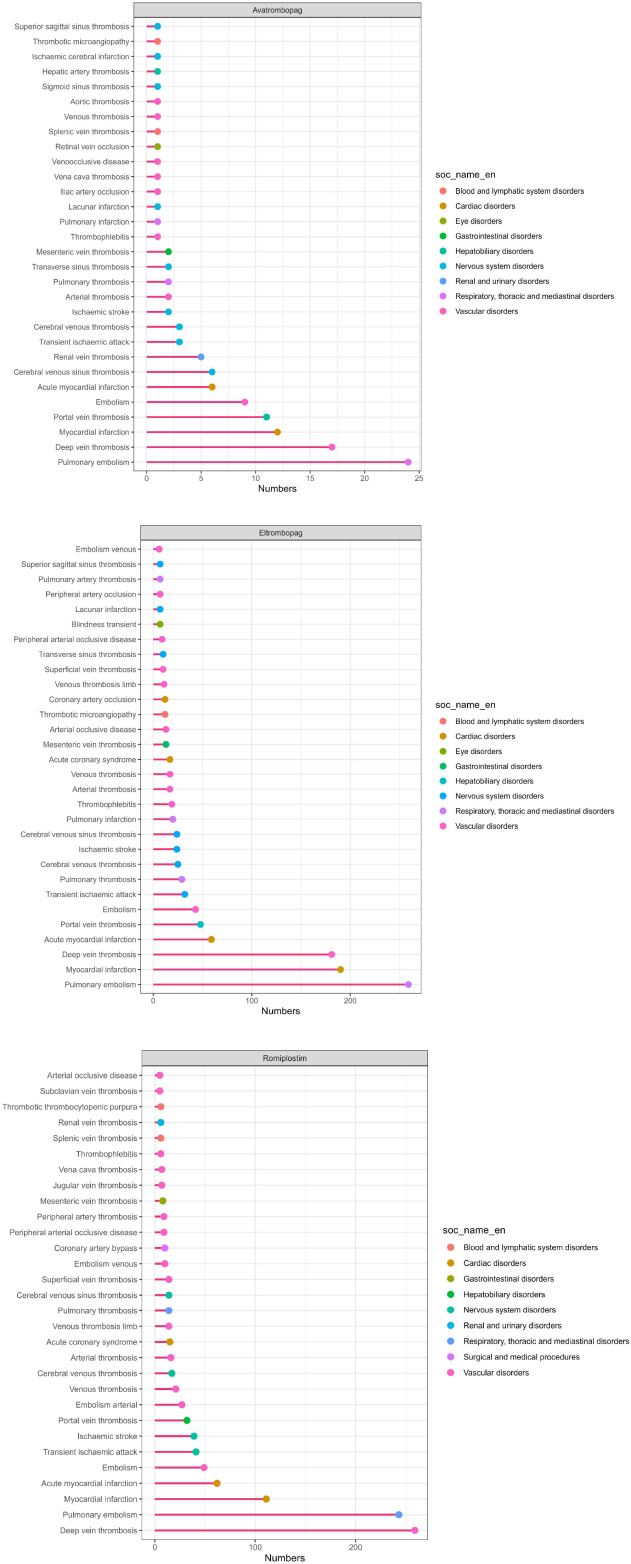
The names and numbers of the top 30 PT with the highest percentage of 3 drug signals detected in the FAERS database and their corresponding SOCs.

This study employed ROR, PRR, and BCPNN methodologies for the analysis of AE signals. A total of 45 TPO-RA signals were obtained following the elimination of input errors, incomplete information, and the screening or exclusion of signals associated with product quality, usage issues, and drug indications. 8, 29, and 37 positive signals were identified for Avatrombopag, Eltrombopag, and Romiplostim (**Tables 4**–**6**). The analysis subsequently concentrated on all signals at the PT level, emphasizing the 30 most frequently occurring signals and those exhibiting the highest signal strength **Figure 2**. The three most commonly reported AEs associated with Avatrombopag were pulmonary embolism, deep vein thrombosis, and portal vein thrombosis. The three AEs with the highest PRR were renal vein thrombosis, portal vein thrombosis, and cerebral venous sinus thrombosis. In the case of Eltrombopag, the three most frequently reported AE signals were pulmonary embolism, deep vein thrombosis, and acute myocardial infarction. The top-ranked PRR were observed for renal embolism, transverse sinus thrombosis, and portal vein thrombosis. regarding romiplostim, the three most frequently reported AE signals were deep vein thrombosis, pulmonary embolism, and acute myocardial infarction, while the top three AE signals by PRR were embolism arterial, retinal artery thrombosis, and splenic vein thrombosis.

**Table 4:** The AEs of Avatrombopag with the highest signal detection, counts in the FAERS database.

No	PT	n	PRR	X^2^	ROR (95% CI)
1	Pulmonary embolism	24	4.24	59.39	4.26 (2.85-6.36)
2	Deep vein thrombosis	17	4.31	43.3	4.33 (2.69-6.97)
3	Portal vein thrombosis	11	58.52	619.27	58.69 (32.43-106.2)
4	Embolism	9	19.06	153.81	19.1 (9.93-36.76)
5	Acute myocardial infarction	6	3.51	10.79	3.52 (1.58-7.83)
6	Cerebral venous sinus thrombosis	6	52.9	304.31	52.98 (23.75-118.19)
7	Renal vein thrombosis	5	136.31	664.92	136.49 (56.53-329.56)
8	Cerebral venous thrombosis	3	26.71	74.09	26.73 (8.61-83.01)

ROR, reporting odds ratio; PRR, proportional reporting ratio; CI, confidence interval; PT, preferred term.

**Table 5:** The AEs of Eltrombopag with the highest signal detection counts in the FAERS database.

No	PT	n	PRR	X^2^	ROR (95% CI)
1	Pulmonary embolism	259	2.92	326.63	2.93 (2.59-3.31)
2	Deep vein thrombosis	181	2.93	230.36	2.94 (2.54-3.4)
3	Acute myocardial infarction	59	2.21	38.81	2.21 (1.71-2.85)
4	Portal vein thrombosis	48	16.51	686.25	16.52 (12.42-21.98)
5	Embolism	43	5.83	171.04	5.84 (4.32-7.88)
6	Pulmonary thrombosis	29	2.73	31.74	2.73 (1.9-3.93)
7	Cerebral venous thrombosis	25	14.39	306.34	14.39 (9.69-21.37)
8	Cerebral venous sinus thrombosis	24	13.64	276.75	13.64 (9.12-20.42)
9	Pulmonary infarction	20	8.52	131.49	8.52 (5.49-13.24)
10	Thrombophlebitis	19	5.88	76.43	5.88 (3.75-9.24)
11	Acute coronary syndrome	17	2.16	10.57	2.16 (1.34-3.48)
12	Arterial thrombosis	17	9.78	132.56	9.79 (6.07-15.79)
13	Venous thrombosis	17	4.6	47.71	4.6 (2.86-7.42)
14	Mesenteric vein thrombosis	13	13.78	151.67	13.78 (7.97-23.84)
15	Venous thrombosis limb	11	5.23	37.39	5.23 (2.89-9.46)
16	Superficial vein thrombosis	10	2.65	10.26	2.65 (1.43-4.93)
17	Transverse sinus thrombosis	10	20.16	177.97	20.17 (10.77-37.75)
18	Peripheral arterial occlusive disease	9	2.18	5.71	2.18 (1.13-4.19)
19	Lacunar infarction	7	3.7	13.74	3.7 (1.76-7.78)
20	Peripheral artery occlusion	7	4.15	16.66	4.15 (1.97-8.72)
21	Pulmonary artery thrombosis	7	10.14	57.02	10.14 (4.81-21.37)
22	Superior sagittal sinus thrombosis	7	9.36	51.7	9.36 (4.44-19.71)
23	Embolism arterial	6	4.74	17.58	4.74 (2.12-10.56)
24	Renal vein thrombosis	6	10.44	50.62	10.44 (4.67-23.36)
25	Splenic vein thrombosis	6	12.69	63.67	12.69 (5.67-28.41)
26	Aortic thrombosis	5	4.93	15.58	4.93 (2.05-11.88)
27	Renal artery thrombosis	4	14.79	50.55	14.79 (5.5-39.73)
28	Renal embolism	4	23.7	84.64	23.7 (8.78-64)
29	Hepatic artery thrombosis	3	12.07	30.04	12.07 (3.86-37.72)

ROR, reporting odds ratio; PRR, proportional reporting ratio; CI, confidence interval; PT, preferred term.

**Table 6:** The AEs of romiplostim with the highest signal detection counts in the FAERS database.

No	PT	n	PRR	X^2^	ROR (95% CI)
1	Deep vein thrombosis	259	7.28	1397.53	7.33 (6.48-8.28)
2	Pulmonary embolism	243	4.74	716.42	4.77 (4.2-5.41)
3	Acute myocardial infarction	62	4.01	139.91	4.02 (3.13-5.16)
4	Embolism	49	11.51	466.88	11.53 (8.7-15.27)
5	Transient ischaemic attack	41	2.38	32.92	2.39 (1.76-3.24)
6	Ischaemic stroke	39	3.94	85.46	3.95 (2.88-5.4)
7	Portal vein thrombosis	32	18.93	536.59	18.94 (13.36-26.85)
8	Embolism arterial	27	37.6	938.34	37.63 (25.68-55.14)
9	Venous thrombosis	21	9.85	166	9.86 (6.42-15.15)
10	Cerebral venous thrombosis	17	16.84	250.52	16.85 (10.45-27.18)
11	Arterial thrombosis	16	15.93	221.46	15.93 (9.73-26.08)
12	Acute coronary syndrome	15	3.3	23.97	3.3 (1.99-5.48)
13	Cerebral venous sinus thrombosis	14	13.68	163.06	13.69 (8.08-23.17)
14	Pulmonary thrombosis	14	2.28	10.03	2.28 (1.35-3.85)
15	Superficial vein thrombosis	14	6.43	63.96	6.43 (3.81-10.88)
16	Venous thrombosis limb	14	11.53	133.67	11.54 (6.82-19.52)
17	Coronary artery bypass	10	2.94	12.81	2.94 (1.58-5.48)
18	Embolism venous	10	5.75	39.08	5.75 (3.09-10.7)
19	Peripheral arterial occlusive disease	9	3.77	18.24	3.77 (1.96-7.25)
20	Peripheral artery thrombosis	9	8.74	61.31	8.74 (4.54-16.83)
21	Mesenteric vein thrombosis	8	14.59	100.27	14.59 (7.27-29.28)
22	Jugular vein thrombosis	7	7.49	39.17	7.49 (3.56-15.74)
23	Vena cava thrombosis	7	7.93	42.19	7.93 (3.77-16.68)
24	Renal vein thrombosis	6	18.07	95.61	18.08 (8.08-40.43)
25	Splenic vein thrombosis	6	21.96	118.3	21.96 (9.81-49.18)
26	Thrombophlebitis	6	3.2	9.05	3.2 (1.44-7.13)
27	Thrombotic thrombocytopenic purpura	6	3.42	10.27	3.42 (1.54-7.63)
28	Subclavian vein thrombosis	5	8.43	32.56	8.43 (3.5-20.31)
29	Pelvic venous thrombosis	4	4.83	12.12	4.83 (1.81-12.9)
30	Peripheral embolism	4	6.31	17.8	6.31 (2.36-16.85)
31	Retinal vein thrombosis	4	12.79	43.12	12.8 (4.78-34.24)
32	Superior sagittal sinus thrombosis	4	9.21	29.1	9.21 (3.45-24.62)
33	Transverse sinus thrombosis	4	13.77	46.92	13.77 (5.14-36.85)
34	Carotid artery thrombosis	3	7.42	16.59	7.42 (2.39-23.08)
35	Cerebral artery thrombosis	3	10.82	26.55	10.82 (3.48-33.7)
36	Postoperative thrombosis	3	9.68	23.2	9.68 (3.11-30.12)
37	Retinal artery thrombosis	3	27.38	74.89	27.39 (8.74-85.8)

ROR, reporting odds ratio; PRR, proportional reporting ratio; CI, confidence interval; PT, preferred term.

### 3.3 The analysis of AEs classified by SOC and ROR signal intensity

AE signals of three drugs were categorized based on the System Organ Classes (SOC) of the impacted organs and systems by utilizing MedDRA version 24.0. In addition, a visual analysis was conducted to investigate PT signals and their associated SOC. Our results showed that vascular disorders represented the primary SOC for the three TPO-RAs in the FAERS database.

**Figure 3:**
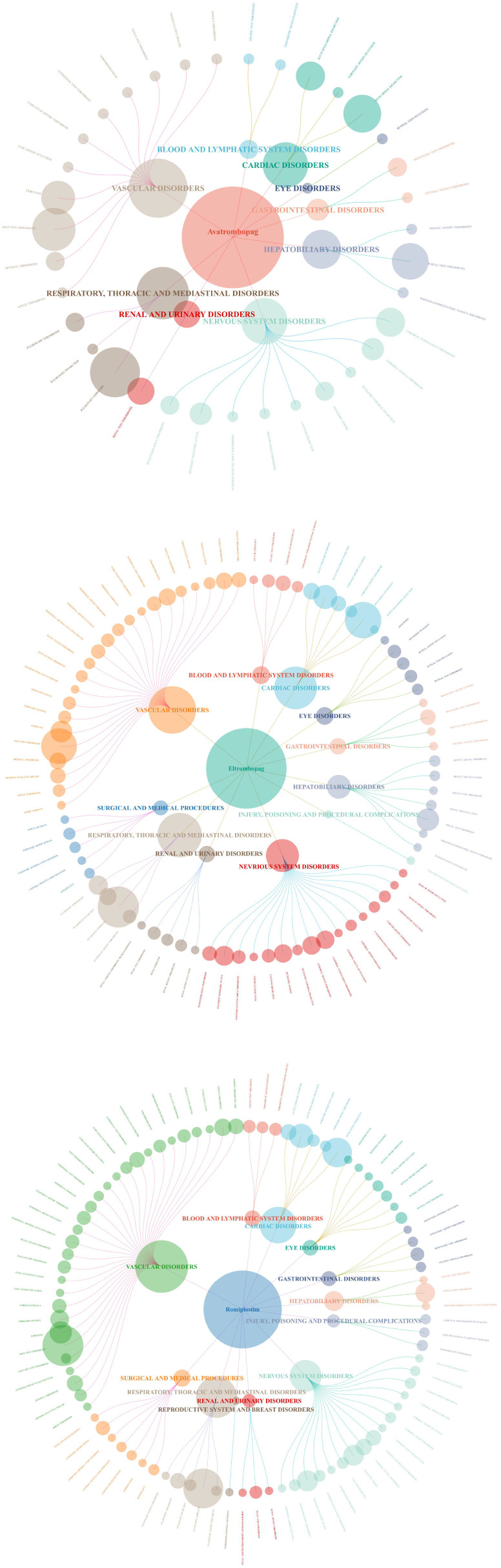
Distribution network plot of AE signals in each SOC for TPO-RA: the root node shows the drug name and AE signal count, with SOCs in the inner ring and AE signal names in the outer ring.

**Figure 4:**
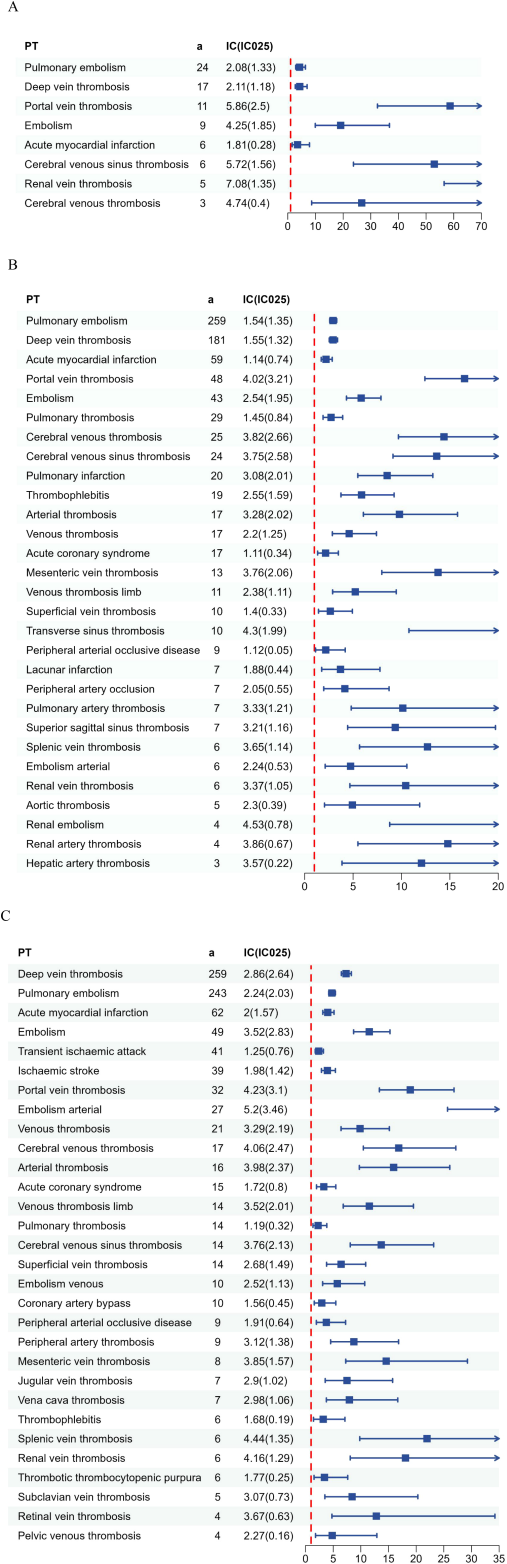
Signal detection results of AE reports and ROR (95% CI). [**(A)** Avatrombopag; **(B)** Eltrombopag; **(C)** Romiplostim]. IC, information component; IC025, the lower limit of 95% CI of the IC; PT, preferred term.

We focused on the ROR with 95% confidence intervals (CI) for the top 30 most common AEs reported for the drugs, enabling visualization through a forest plot (**Figure 3**). In relation to Avatrombopag, the most significant signal identified was Renal Vein Thrombosis, exhibiting a (ROR: 136.49, CI: 56.53-329.56), categorized under renal and urinary disorders. Portal vein thrombosis has been observed with a (ROR: 58.69, CI: 32.43-106.2), classified under hepatobiliary disorders. For Eltrombopag, the strongest signal was renal embolism, with an (ROR: 23.7, CI: 8.78-64), belonging to renal and urinary disorders. The highest AE associated with Romiplostim was embolism arterial, with an (ROR: 37.63, CI: 25.68 - 55.14) (**Figure 4**).

### 3.4 TTO analysis of AEs based on TPO-RA

The largest proportion of adverse events associated with TPO-RA manifested within the first 30 days following the commencement of treatment (**Table 7**). The mean TTO of AEs was 229.5 days. Quartile distribution showed that the TTO was ≤25 days in 25% of patients, with a median of 81 days, and ≤263 days in 75% of patients. The interquartile range was 238 days.

**Table 7:** TTO of TPO-RA-related AEs.

Group	n	Percent
0–30 days	282	30.92%
31–60 days	149	16.34%
61–90 days	93	10.20%
91–120 days	67	7.34%
121–150 days	40	4.39%
151–180 days	37	4.06%
181–360 days	104	11.40%
>360 days	140	15.35%

### 3.5 Investigation of factors associated with TPO-RA-related thromboembolic events using multivariate logistic regression

Multivariate logistic regression analysis demonstrated that patients with advanced age were significantly more likely to experience thromboembolic events. Univariate logistic regression analysis was conducted to further examine the factors associated with thromboembolic events, employing the previously described method. Significant differences were observed in factors including age, weight, gender, follow-up duration, and drug type. The univariate analysis revealed that, in comparison to the <18-year-old cohort, the 18-65-year-old group (OR: 4.86, 95% CI: 4.12-5.78), the 65-85-year-old group (OR: 6.95, 95% CI: 5.89-8.28), and the >85-year-old group (OR: 13.96, 95% CI: 11.71-16.75) all exhibited a significantly heightened reporting signals of thromboembolic events. Individuals’ weight >100kg (OR: 1.58, 95% CI: 1.45-1.72) also presented an increased reporting signals, whereas those in the 50-100kg range (OR: 0.87, 95% CI: 0.83-0.92) showed a diminished reporting signal. Male had a lower reporting signals compared to female (OR: 0.81, 95% CI: 0.77-0.84). The cohort with a follow-up duration exceeding 730 days demonstrated an increased reporting signal (OR: 1.32, 95% CI: 1.21-1.45). Patients treated with Eltrombopag (OR: 0.42, 95% CI: 0.34-0.53) and Romiplostim (OR: 0.30, 95% CI: 0.24-0.37) exhibited a significantly lower reporting signals compared to those receiving Avatrombopag (**Table 8**).

**Table 8:** Univariable logistic regression analysis: odds ratios for thromboembolic AEs associated with TPO-RA.

Dependent: y		No_target	Target	OR (univariable)	OR	OR_CI	OR_UI
AGE	<18	4011 (96.6)	143 (3.4)	–			
	18-65	27788 (85.2)	4812 (14.8)	4.86 (4.12-5.78, *p* < 0.001^***^)	4.86	4.12	5.78
	65-85	19174 (80.1)	4753 (19.9)	6.95 (5.89-8.28, *p* < 0.001^***^)	6.95	5.89	8.28
	>85	2832 (66.8)	1409 (33.2)	13.96 (11.71-16.75, *p* < 0.001^***^)	13.96	11.71	16.75
WT	<50	11839 (82.3)	2542 (17.7)	–			
	50-100	38303 (84.2)	7169 (15.8)	0.87 (0.83-0.92, *p* < 0.001^***^)	0.87	0.83	0.92
	>100	2807 (74.6)	955 (25.4)	1.58 (1.45-1.72, *p* < 0.001^***^)	1.58	1.45	1.72
SEX	Female	32733 (81.7)	7315 (18.3)	–			
	Male	21016 (84.7)	3799 (15.3)	0.81 (0.77-0.84, *p* < 0.001^***^)	0.81	0.77	0.84
TIME	<365	48825 (83.1)	9943 (16.9)	–			
	365-730	2696 (82.9)	558 (17.1)	1.02 (0.92-1.12, p=0.735)	1.02	0.92	1.12
	>730	2284 (78.8)	616 (21.2)	1.32 (1.21-1.45, *p* < 0.001^***^)	1.32	1.21	1.45
DRUG	Avatrombopag	218 (63.2)	127 (36.8)	–			
	Eltrombopag	24122 (80.3)	5924 (19.7)	0.42 (0.34-0.53, *p* < 0.001^***^)	0.42	0.34	0.53
	Romiplostim	29465 (85.3)	5066 (14.7)	0.30 (0.24-0.37, *p* < 0.001^***^)	0.3	0.24	0.37

*** (*p* <.001).

OR (univariable), crude odds ratio from univariable logistic regression; OR, adjusted odds ratio from multivariable logistic regression; OR_CI, lower limit of the 95% confidence interval of OR; OR_UI, upper limit of the 95% confidence interval of OR.

Multivariate logistic regression confirmed that advanced age remained strongly associated with increased reporting signals of thromboembolic events after adjusting for the possible confounding factors. The highest reporting signals was in the >85 years group (OR: 14.94, 95% CI: 12.51–17.97). In contrast, patients who were treated with Eltrombopag (OR: 0.32, 95% CI: 0.25-0.40) or Romiplostim (OR: 0.20, 95% CI: 0.16-0.25) were at decreased reporting signals of developing thromboembolic events (**Table 9**).

**Table 9:** Multivariable logistic regression analysis: odds ratios for thromboembolic AEs associated with TPO-RA.

Dependent: y		No_target	Target	OR (multivariable)	OR	OR_CI	OR_UI
AGE	<18	4011 (96.6)	143 (3.4)	–			
	18-65	27788 (85.2)	4812 (14.8)	5.05 (4.26-6.04, *p* < 0.001^***^)	5.05	4.26	6.04
	65-85	19174 (80.1)	4753 (19.9)	7.68 (6.48-9.18, *p* < 0.001^***^)	7.68	6.48	9.18
	>85	2832 (66.8)	1409 (33.2)	14.94 (12.51-17.97, *p* < 0.001^***^)	14.94	12.51	17.97
WT/kg	<50	11839 (82.3)	2542 (17.7)	–			
	50-100	38303 (84.2)	7169 (15.8)	0.78 (0.74-0.82, *p* < 0.001^***^)	0.78	0.74	0.82
	>100	2807 (74.6)	955 (25.4)	1.50 (1.37-1.64, *p* < 0.001)	1.5	1.37	1.64
SEX	Female	32733 (81.7)	7315 (18.3)	–			
	Male	21016 (84.7)	3799 (15.3)	0.89 (0.85-0.93, *p* < 0.001^***^)	0.89	0.85	0.93
TIME	<365	48825 (83.1)	9943 (16.9)	–			
	365-730	2696 (82.9)	558 (17.1)	1.08 (0.98-1.19, *p* = 0.099)	1.08	0.98	1.19
	>730	2284 (78.8)	616 (21.2)	1.38 (1.26-1.52, *p* < 0.001^***^)	1.38	1.26	1.52
DRUG	Avatrombopag	218 (63.2)	127 (36.8)	–			
	Eltrombopag	24122 (80.3)	5924 (19.7)	0.32 (0.25-0.40, *p* < 0.001^***^)	0.32	0.25	0.4
	Romiplostim	29465 (85.3)	5066 (14.7)	0.20 (0.16-0.25, *p* < 0.001^***^)	0.2	0.16	0.25

*** (*p* <.001).

OR (univariable), crude odds ratio from univariable logistic regression; OR, adjusted odds ratio from multivariable logistic regression; OR_CI, lower limit of the 95% confidence interval of OR; OR_UI, upper limit of the 95% confidence interval of OR.

## 4 Discussion

In this FAERS-based pharmacovigilance study, we characterized thromboembolic adverse event signals associated with three approved TPO-RA used for ITP. Overall, venous thromboembolism signals predominated across agents, while the anatomic distribution of reported thromboembolic events differed by drug, suggesting heterogeneity in real-world safety profiles.

As an important option for the second-line treatment of ITP, TPO-RA has been proven to effectively increase platelet counts and reduce bleeding events (22). However, real-world data on its thromboembolic risk remain limited, which highlights the clinical relevance of the findings from this study. TPO-RAs promote platelet production by activating the TPO receptor on megakaryocytes, but it may also modify platelet function and coagulation balance, potentially elevating the risk of thrombosis (23).

### 4.1 Analysis of specific thromboembolic AEs

Pulmonary Embolism and Deep Vein Thrombosis were the main types of thromboembolic events common to the three TPO-RAs, which was consistent with previous studies. The elevated occurrence of venous thrombosis events during TPO-RA treatment might be attributed to various mechanisms. TPO-RA significantly enhanced platelet counts, especially during the initial phase of treatment. The swift elevation in platelet counts might result in heightened blood viscosity and an increased propensity for thrombosis. Portal vein thrombosis was notably prominent in patients treated with Avatrombopag (ROR: 58.69). This finding required specific consideration. Portal Vein Thrombosis is a relatively rare but serious thromboembolic complication, commonly observed in patients with liver cirrhosis, malignant tumors, or hematological diseases (4).

Acute myocardial infarction was frequently reported with Eltrombopag and Romiplostim, suggesting a potential arterial thromboembolic risk. Patients with ITP may have an intrinsically increased thromboembolic tendency due to disease-related factors such as young hyperactive platelets, platelet microparticles, antiphospholipid antibodies, and reduced natural anticoagulant activity (24). Cerebrovascular events (e.g., cerebral vascular thrombosis and transient ischemic attack) were reported for all three drugs; although reported less often, their clinical severity warrants vigilance.

Renal Vein Thrombosis exhibited a markedly high signal intensity in the Avatrombopag group (ROR: 136.49), indicating that Avatrombopag may be associated with an unusual thromboembolic risk. Although Renal Vein Thrombosis is rare, severe renal function impairment can occur. Such specificity may be an indication of the pharmacokinetic properties or receptor-binding specificity of Avatrombopag, and further mechanistic studies are necessary to account for this.

### 4.2 Risk factors and pathogenesis

This study demonstrated that advanced age was a significant risk factor for TPO-RA-associated thromboembolic events. Elderly patients have concurrent thromboembolic risk factors like cardiovascular disease, diabetes, and hypertension that may act synergistically with prothrombotic behavior of TPO-RA (25). PAI-1 is typically elevated in the elderly, and it is a good predictor of impairment in the fibrinolytic system (26). Prolonged PAI-1 levels, as the key inhibitor of the fibrinolytic pathway, result in decreased fibrinolytic activity and decreased capacity for thrombus dissolution, enhancing thromboembolic susceptibility (27). Levels of PAI-1 were also elevated in TPO-RA-treated ITP patients, predisposing to the formation of more fibrinolytic-resistant thrombi. This mechanism may be of key importance in the enhanced thrombosis risk observed in older patients (28).

Patients who have body weight >100 kg were at very high reporting odds for thrombosis, which is closely associated with obesity-related pathophysiologic mechanisms participating in thrombogenesis. Obese patients have common metabolic derangements in the form of elevated PAI-1 levels, excessive production of inflammatory factors, and insulin resistance, all of which participate in thrombosis development (29). Also, the platelet function in obese patients may undergo alterations in the form of enhanced platelet aggregability and over-release of microparticles (30, 31).

Patients with a follow-up duration exceeding 730 days exhibited a 32% increased risk of thrombosis, indicating that prolonged TPO-RA treatment may correlate with cumulative thromboembolic risk. This finding aligns with the EXTEND study results, which revealed a significant time-dependent pattern in the thromboembolic risk of Eltrombopag (32). The increased thromboembolic risk associated with long-term treatment may stem from several mechanisms: persistent platelet activation, chronic changes in the coagulation-anticoagulation equilibrium, and enduring effects on vascular endothelial function.

### 4.3 Exploration of molecular mechanisms

The increased thrombogenicity associated with TPO-RAs is likely multifactorial and cannot be explained solely by elevated platelet counts. Beyond stimulating megakaryopoiesis, thrombopoietin signaling may prime platelet reactivity, lowering the activation threshold and enhancing aggregation responses, thereby facilitating thromboembolic events in susceptible patients (33). Mechanistically, studies have demonstrated that TPO-RA exposure is associated with a procoagulant platelet phenotype, including increased platelet apoptosis and phosphatidylserine exposure, which provides a catalytic surface for prothrombin complex binding and thrombin generation (34, 35). In parallel, higher platelet PAI-1 content following TPO-RA treatment renders thrombi more resistant to fibrinolysis, favoring thrombus persistence (28). Elevated circulating platelet-derived microparticles, which exhibit enhanced procoagulant activity, may further amplify coagulation and thromboinflammatory signaling (36, 37).

In addition, increased expression of von Willebrand factor promotes platelet adhesion to the vascular endothelium (38), while an imbalance in the angiopoietin-2/angiopoietin-1 axis contributes to endothelial dysfunction and vascular instability. Importantly, ITP itself is increasingly recognized as a disorder characterized by a baseline prothrombotic milieu, involving young hyperreactive platelets, circulating microparticles, antiphospholipid antibodies, and reduced natural anticoagulant activity; TPO-RAs may further shift this fragile hemostatic balance toward thrombosis (39–41). Collectively, these mechanisms provide a biologically plausible explanation for the thromboembolic signals observed in randomized trials and meta-analyses, as well as the time-dependent increase in thromboembolic events reported with prolonged TPO-RA exposure in long-term extension studies (42–44).

### 4.4 Clinical implications and therapeutic recommendations

Given the signal-detection nature of this FAERS analysis, a baseline evaluation of thromboembolic risk may be considered prior to initiating TPO-RA therapy. These considerations are hypothesis-generating and intended to raise clinical awareness rather than provide practice-changing management recommendations. Heightened clinical vigilance may be warranted, particularly in patients who are older (e.g., >85 years), have obesity (BMI >30 kg/m² or body weight >100 kg), or have a prior history of thrombosis. Where clinically indicated and consistent with local practice, baseline coagulation assessment may be reasonable for patients at higher thromboembolic risk. This assessment may include thrombotic markers such as D-dimer, PAI-1 levels, and von Willebrand factor. TPO-RA use may warrant additional caution in ITP patients with antiphospholipid antibodies, given the established thrombotic propensity associated with antiphospholipid antibodies (45, 46). Any consideration of prophylactic anticoagulation should be individualized and guideline-informed, with careful balancing of thrombotic and bleeding risks; importantly, this FAERS-based signal-detection analysis does not support routine prophylactic anticoagulation.

For patients receiving longer-term TPO-RA therapy, ongoing clinical vigilance for thromboembolic events may be prudent, and monitoring strategies should be tailored to individual risk profiles and require prospective validation. Regular monitoring of platelet counts is advised to avoid excessive elevation of platelet counts (>400 × 10^9^/L). Where clinically justified, periodic assessment of selected thrombotic markers (e.g., D-dimer, PAI-1, and von Willebrand factor) may be considered. Patient education regarding early symptoms suggestive of thromboembolic events (e.g., lower extremity swelling/pain, chest pain, dyspnea, or sudden headache) may facilitate timely medical evaluation.

## 5 Limitations

This study presents several limitations. First, the FAERS database might not include all patients receiving TPO-RA therapy, which could result in underreporting or selective reporting bias. Due to the nature of FAERS as a spontaneous reporting system, the incidence of serious AEs might exceed that of less severe events. Moreover, FAERS lacks denominators and exposure information (e.g., the number of treated patients and treatment duration), so incidence rates and comparative risks cannot be estimated; disproportionality results should be interpreted as signals rather than causal effects. Variations in the timing of market launches and the market share of different drugs may influence the comparison of report quantities. Second, the FAERS database documents reports of AEs instead of data derived from rigorously controlled clinical trials. Reporting volume also reflects time on market and clinical uptake; notably, Avatrombopag received later approvals (and may have shorter cumulative exposure time) than Eltrombopag and Romiplostim, which may contribute to fewer FAERS reports and limits cross-agent comparisons. Indication bias is possible, particularly because Eltrombopag has non-ITP indications (and may be used in broader patient populations), which could introduce heterogeneity in baseline thromboembolic risk across reports. Therefore, the observed association between thromboembolic events and TPO-RA use may be affected by confounding factors, including patients’ underlying diseases, concomitant medications, and lifestyle factors.

## 6 Conclusions

TPO-RA has been widely used as a second-line treatment for ITP, but real-world data on its thromboembolic AEs remain limited. This study addressed this critical gap in safety surveillance. A systematic analysis based on the FAERS database showed that TPO-RA-related thromboembolic events were prevalent in clinical practice, with 2,092 thromboembolic reports identified. There were significant differences in the range of thromboembolic events with various drugs: Avatrombopag was most frequently linked to Pulmonary Embolism, Deep Vein Thrombosis, and Portal Vein Thrombosis, while Eltrombopag and Romiplostim were most frequently reported with Pulmonary Embolism, Deep Vein Thrombosis, and Acute Myocardial Infarction. Reports involving patients aged >85 years and those with long treatment duration (>730 days) showed higher reporting odds of thromboembolic events. The findings provide essential advice for individualized management of TPO-RA therapy: elderly patients require more monitoring and surveillance of thrombosis, especially vigilant monitoring in the initial phases of treatment. Implementing a standardized system for thrombosis risk assessment and monitoring strategies is essential for enhancing treatment safety.

## Data Availability

The raw data supporting the conclusions of this article will be made available by the authors, without undue reservation.
